# Association Between Severity and the Determinant-Based Classification, Atlanta 2012 and Atlanta 1992, in Acute Pancreatitis: A Clinical Retrospective Study

**DOI:** 10.1097/MD.0000000000000638

**Published:** 2015-04-03

**Authors:** Yuhui Chen, Lu Ke, Zhihui Tong, Weiqin Li, Jieshou Li

**Affiliations:** Department of General Surgery, Jinling Hospital, Nanjing University School of Medicine, Nanjing, People's Repubic of China.

## Abstract

Recently, the determinant-based classification (DBC) and the Atlanta 2012 have been proposed to provide a basis for study and treatment of acute pancreatitis (AP). The present study aimed to evaluate the association between severity and the DBC, the Atlanta 2012 and the Atlanta 1992, in AP.

Patients admitted to our center with AP from January 2007 to July 2013 were reviewed retrospectively. Patients were assigned to severity categories for all the 3 classification systems. The primary outcomes include long-term clinical prognosis (mortality and length-of-hospital stay), major complications (intraabdominal hemorrhage, multiple-organ dysfunction, single organ failure [OF], and sepsis) and clinical interventions (surgical drainage, continuous renal replace therapy [CRRT] lasting time, and mechanical ventilation [MV] lasting time). The classification systems were validated and compared in terms of these abovementioned primary outcomes.

A total of 395 patients were enrolled in this retrospective study with an overall 8.86% in-hospital mortality. Intraabdominal hemorrhage was present in 27 (6.84%) of the patients, multiple-organ dysfunction in 73(18.48%), single OF in 67 (16.96%), and sepsis in 73(18.48%). For each classification system, different categories regarding severity were associated with statistically different clinical mortality, major complications, and clinical interventions (*P* < 0.05). However, the Atlanta 2012 and the DBC performed better than the Atlanta 1992, and they were comparable in predicting mortality (area under curve [AUC] 0.899 and 0.955 vs 0.585, *P* < 0.05); intraabdominal hemorrhage (AUC 0.930 and 0.961 vs 0.583, *P* < 0.05), multiple-organ dysfunction (AUC 0.858 and 0.881 vs 0.595, *P* < 0.05), sepsis (AUC 0.826 and 0.879 vs 0.590, *P* < 0.05), and surgical drainage (AUC 0.900 and 0.847 vs 0.606, *P* < 0.05). For continuous variables, the Atlanta 2012 and the DBC were also better than the Atlanta 1992, and they were similar in predicting CRRT lasting time (Somer D 0.379 and 0.360 vs 0.210, *P* < 0.05) and MV lasting time (Somer D 0.344 and 0.336 vs 0.186, *P* < 0.05).

All the 3 classification systems accurately classify the severity of AP. However, the Atlanta 2012 and the DBC performed better than the Atlanta 1992, and they were comparable in predicting long-term clinical prognosis, major complications, and clinical interventions.

## INTRODUCTION

Acute pancreatitis (AP) is a common and potentially lethal disease with wide clinical variations, ranging from mild abdominal discomfort to severe form with high morbidity or even mortality.^1–4^ To provide a basis for international communication and treatment of AP, it is of great importance to classify the severity of AP into subgroups accurately. The Atlanta 1992,^5^ a classification system recommended by 41 recognized experts in AP, was widely used for over 20 years. It provides clear clinical definitions for AP and its complications, as well as a better practice system of management. However, based on the advancements in the understanding of pathophysiology and the natural course of AP, several authors had suggested the need for a revision of the Atlanta 1992 in the last decades to provide new directions for research and improve clinical practice.^6–10^

In 2013, the determinant-based classification (DBC) and the Atlanta 2012 have been proposed,^11,12^ and both the 2 systems were led by international groups of experts that utilized a web-based consensus-building approach. According to the DBC, patients were assigned to 4 grades of severity based on the presence of (peri)pancreatic necrosis (PN) and transient/persistent organ failure (OF) or not. It presumes 2 “causal association” with severity, called “determinants”: “(peri)pancreatic necrosis” and “organ failure.” However, the other factors such as obesity^13^ may also influence the clinical outcomes, so the association between the DBC and disease severity needs more evidence. When it comes to the Atlanta 2012, it assigned patients 3 grades of severity. Severe AP was defined if persistent OF was present; moderately severe AP was defined if acute fluid collection, PN, pseudocyst, pancreatic abscess, exacerbation of preexisting comorbidity or transient OF were present; whereas mild AP was defined if none of the complications mentioned above was present. In this classification, persistent OF was the most important “causal association” with severity.

In order to standardize the classification systems internationally and provide a better understanding of the disease, it is of great importance to compare these classification systems. However, to the best of our knowledge, few data was found in this point. The aim of this study is to compare the association between different classification systems and disease severity.

## METHODS

### Patients

From January 2007 to July 2013, a consecutive series of patients with AP admitted to the surgical intensive care unit (SICU) of the institute of General Surgery, Jinling Hospital, Nanjing, China, within 7 days from the onset of the disease were studied. Diagnosis of AP was based on abdominal pain suggesting AP, serum amylase at least 3 times the upper limit of normal, or computed tomography according to the Atlanta criteria. Patients were excluded if there was a known history of AP, cancer on admission or age younger than 18. Moreover, patients were excluded if they quit the therapy because of nonmedical reason or were transferred to other hospitals before full recovery. Finally, patients who were lost to follow-up were excluded as well. This retrospective study was approved by the Medicine Institutional Review Board of Jinling Hospital. All patients provided informed consent to participate in the study.

### Management

All patients received standard medical treatment including continuous hemodynamic monitor, fluid resuscitation, early enteral nutrition, prophylactic antibiotics in patients with severe AP according to the Atlanta 1992, and so on. Infected pancreatic necrosis (IPN) was treated using minimally invasive and surgical drainage. Our criteria for transferring patients from SICU to general wards are patients without any of the followings: OF, uncontrolled intraabdominal hemorrhage, and severe infectious complications such as sepsis and unstable hemodynamics. After hospital discharge, patients were required to come back to our hospital for recheck (computed tomography and other test when necessary) every month in the first 2 months and every 2 months in the following 10 months. This period of time will be prolonged if needed. Those who did not come back to our hospital for further examination would be followed through Internet and/or cell phone.

### Grouping Criteria

According to the Atlanta 1992, patient was defined as severe AP if local complications (acute fluid collection, PN, pseudocyst, and pancreatic abscess) or OF developed or acute physiology and chronic health evaluation scoring system (APACHE II) score ≥8 or Ranson score ≥3, whereas mild AP was defined if none of these was present.

Patients were assigned to 4 grades of severity of AP based on the presence of (peri)PN and transient/persistent OF or not according to the DBC. Critical AP was defined when infected (peri)PN and persistent OF were present together; severe AP was defined when infected (peri)PN or persistent OF development; moderate AP was defined when sterile PN or/and transient OF were present; whereas mild AP was defined when there was no (peri)PN, as well as OF in contrast.

The Atlanta 2012 assigned patients to 3 grades of severity. Severe AP was defined if persistent OF was present; moderately severe AP was defined if acute fluid collection, PN, pseudocyst, pancreatic abscess, exacerbation of preexisting comorbidity or transient OF was present; whereas mild AP was defined if none of the complications mentioned above was present.

### Definitions

The definitions of organ dysfunction were based on a score of ≥2 in the sequential organ failure assessment (SOFA) scoring system.^14^ More specifically, transient OF was defined as OF involving the respiratory, cardiovascular, or renal systems lasting <48 hours, whereas persistent OF was defined as OF in any of the 3 organ systems lasting ≥48 hours.^11,12^ Multiple-organ dysfunction syndrome (MODS) was defined as the combined dysfunction of 2 major organ systems.^15^ Sepsis was defined when patients developed both systemic inflammatory response syndrome and infection according to the Surviving Sepsis Campaign Guidelines Committee.^16^ Cardiovascular dysfunction was defined if mean arterial pressure (MAP) was <65 mm Hg after adequate fluid resuscitation or vasoactive agents was needed to maintain MAP ≥65 mm Hg. PN was diagnosed according to the results of contrast-enhanced computed tomography performed at least 48 hours after the onset of the disease. Our criteria for the diagnosis of pancreatic infection are as follows: positive findings in bacterial culture of abdominal fluid and repeated temperature increases.

### Data Collection and Outcome Measures

Baseline data included age, sex, etiology, the SOFA score, Ranson score, and the APACHE II score. The primary data included long-term clinical prognosis (mortality and length-of-hospital stay [LOS]), major complications (intraabdominal hemorrhage, multiple-organ dysfunction, single OF, and sepsis), and clinical interventions (surgical drainage, continuous renal replace therapy [CRRT] lasting time, and mechanical ventilation [MV] lasting time).

### Statistical Analysis

Data were analyzed using SPSS 17.0 for Windows (SPSS, Chicago, IL). All statistical tests were 2-tailed, and significance level was set at *P* < 0.05. Pairwise testing between severity grades within a classification system was performed using Fisher exact for binary outcomes and were expressed as percentage, whereas Kruskal–Wallis tests were used for continuous outcomes and expressed as mean (the 25% percentile to the 75% percentile). The area under the receiver operating characteristic curve was used for binary outcomes to describe the predictive value of a classification system, whereas the Somer D was reported for continuous outcomes. Pairwise comparison of area under curve (AUC) value and Somer D value was performed with Sidak adjustment to control for type I error. Logistic regression test was performed to analyze risk factors for hospital mortality.

## RESULTS

A total of 395 patients were enrolled in this retrospective study with an overall 8.86% in-hospital mortality (Table 1). Intraabdominal hemorrhage was present in 27 (6.84%) of the study patients, MODS in 73 (18.48%), single OF in 67 (16.96%), and sepsis in 73 (18.48%).

**TABLE 1:**
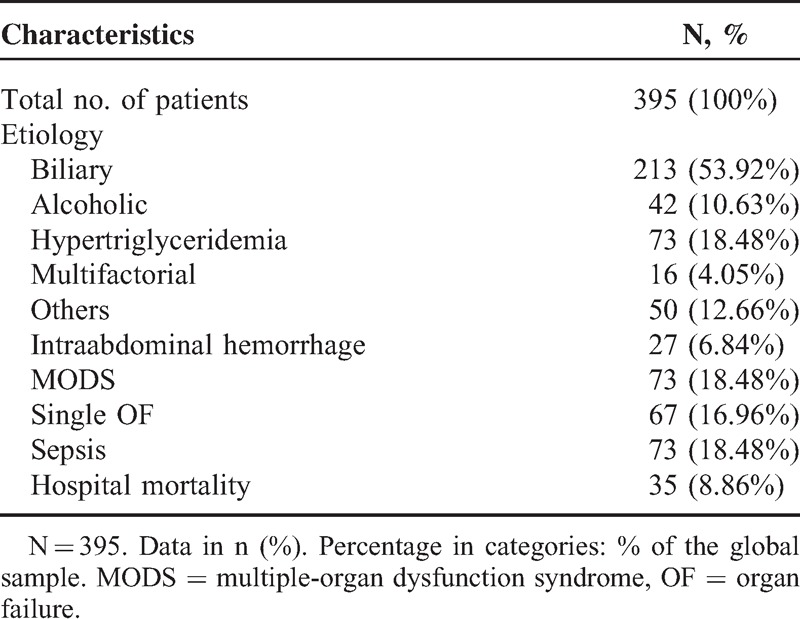
Patient Characteristics

According to the Atlanta 1992, 61 patients were defined as mild AP whereas the other 334 were defined as severe AP. No significant difference was found between the 2 groups in age and gender (Table 2). The Atlanta 1992 classification accurately classify the severity of AP into subgroups evidenced by significantly different clinical outcomes including development of PN (72% vs 0%, *P* < 0.001), IPN (21% vs 0%, *P* < 0.001), intraabdominal hemorrhage (8% vs 0%, *P* = 0.013), MODS (22% vs 0%, *P* < 0.001), single OF (20% vs 0%, *P* < 0.001), and sepsis (22% vs 0%, *P* < 0.001). Moreover, patients with severe AP also showed longer CRRT lasting time (1.89 (0–0) vs 0 (0–0) day, *P* < 0.001; Table 3), MV lasting time (1.22 (0–0) vs 0 (0–0) day, *P* < 0.001), and LOS (14.10 (6–12) vs 5.20 (4–6.5) day, *P* < 0.001), as well as higher rates of surgical drainage (32% vs 0%, *P* < 0.001) and hospital death (10% vs 0%, *P* = 0.005; Table 4).

**TABLE 2:**
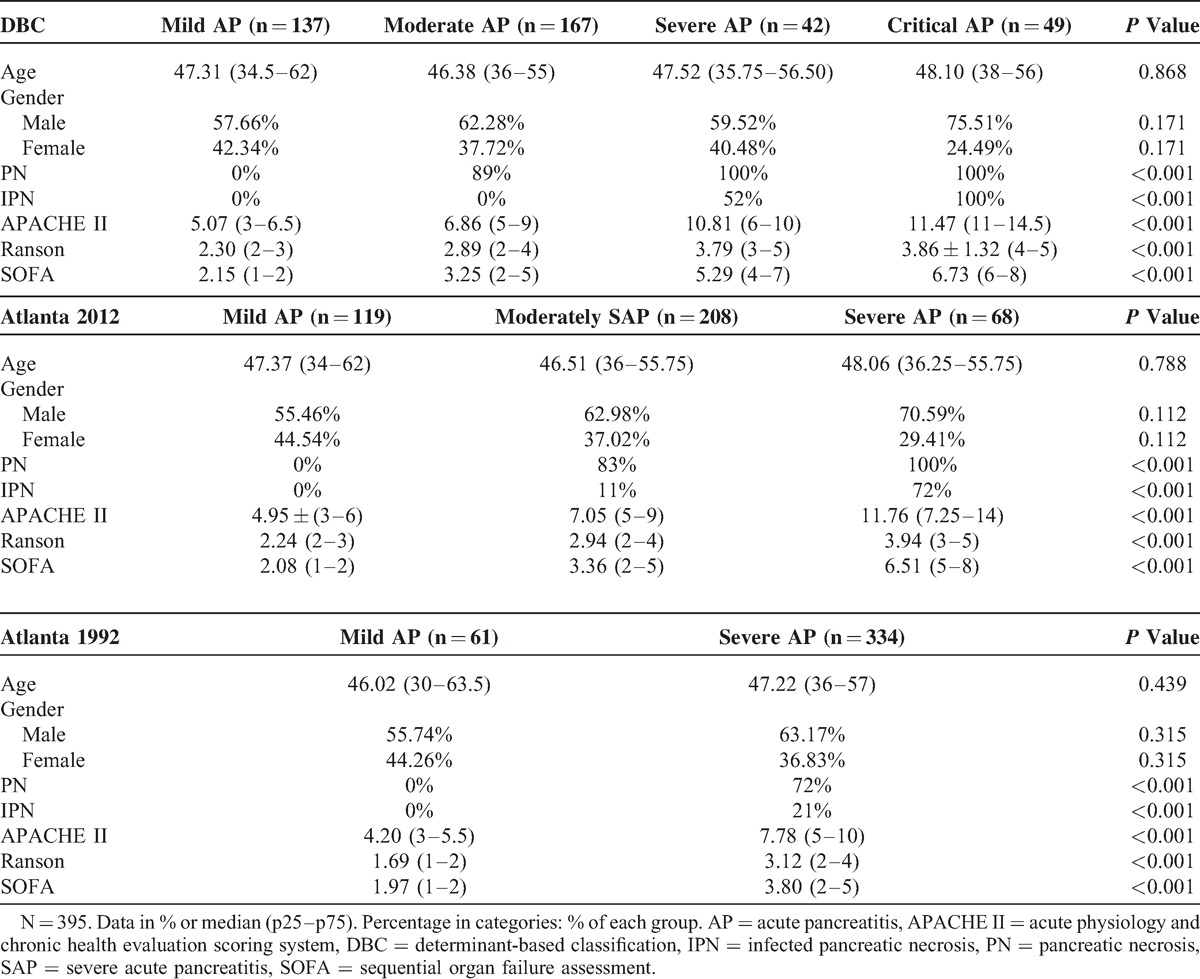
Clinical Characteristic of Different Classification Systems

**TABLE 3:**
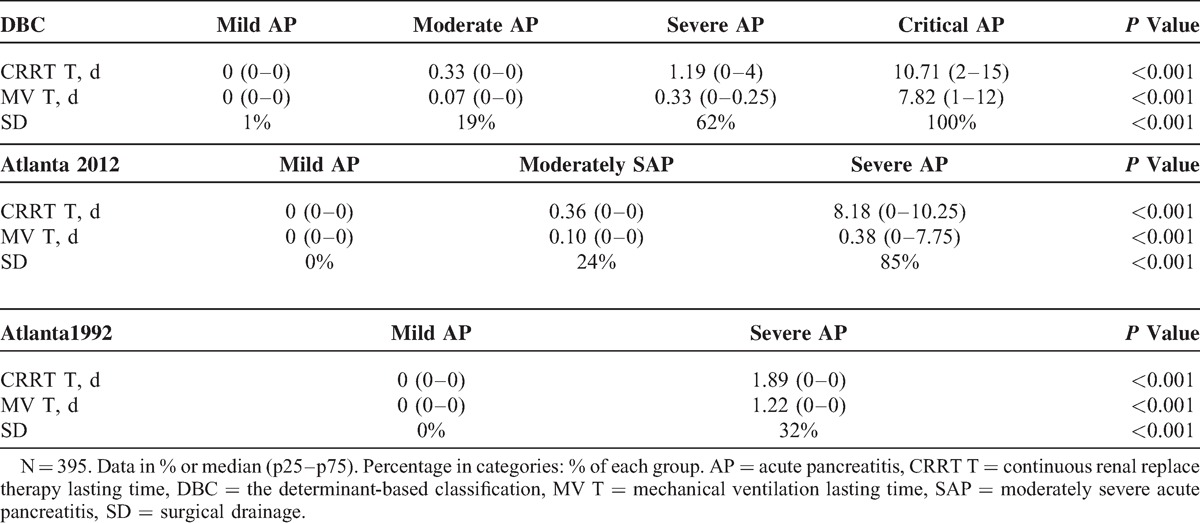
Major Therapies of Different Classification Systems

**TABLE 4:**
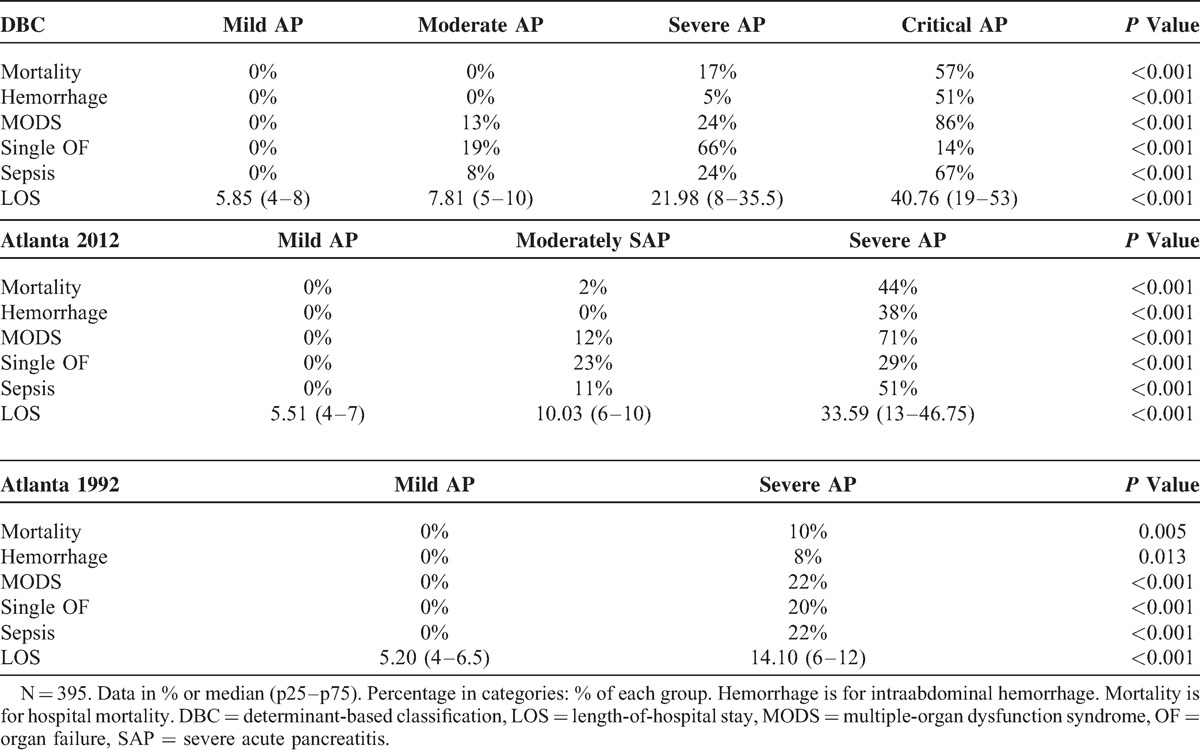
Primary Clinical Outcomes of Different Classification Systems

On the basis of the Atlanta 2012, 68 patients were defined as severe AP, 208 patients were defined as moderately severe AP, whereas the other 119 patients were defined as mild AP. A total of 137 patients were defined as mild AP according to the DBC, whereas 167 were defined as moderate AP, 42 were defined as severe AP, whereas the other 49 patients were defined as critical AP. No significant difference was found among the different groups in age and gender (Table 2). Both the Atlanta 2012 and the DBC accurately classify the severity of AP in subgroups evidenced by significantly different clinical outcomes including development of PN (*P* < 0.001), IPN (*P* < 0.001, Table 2), intraabdominal hemorrhage (*P* < 0.001), MODS (*P* < 0.001), single OF (*P* < 0.001), and sepsis (*P* < 0.001, Table 4); and significantly longer CRRT lasting time (*P* < 0.001, Table 3), MV lasting time (*P* < 0.001), and LOS (*P* < 0.001, Table 4), as well as higher rates of surgical drainage (*P* < 0.001, Table 3) and hospital death (*P* < 0.001).

However, the Atlanta 2012 and the DBC performed better than the Atlanta 1992, and they were comparable in predicting mortality (AUC 0.899 and 0.955 vs 0.585, *P* < 0.05, Table 5), intraabdominal hemorrhage (AUC 0.930 and 0.961 vs 0.583, *P* < 0.05), multiple-organ dysfunction (AUC 0.858 and 0.881 vs 0.595, *P* < 0.05), sepsis (AUC 0.826 and 0.879 vs 0.590, *P* < 0.05), and surgical drainage (AUC 0.900 and 0.847 vs 0.606, *P* < 0.05). For continuous variables, both the Atlanta 2012 and the DBC were better than the Atlanta 1992, and they were similar in predicting CRRT lasting time (Somer D 0.379 and 0.360 vs 0.210, *P* < 0.05) and MV lasting time (Somer D 0.344 and 0.336 vs 0.186, *P* < 0.05).

**TABLE 5:**
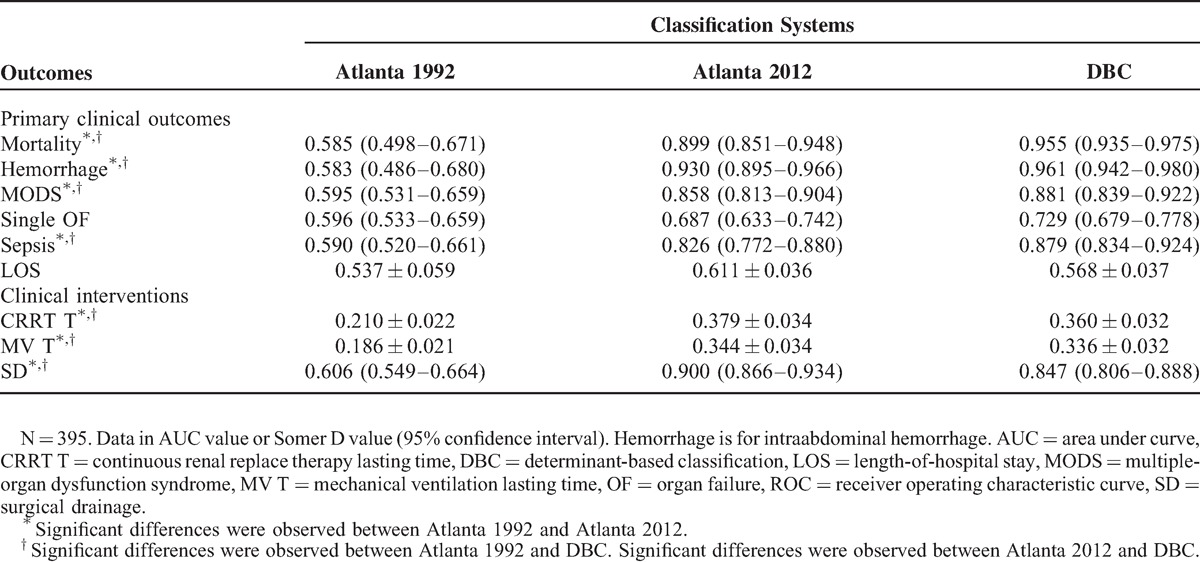
Comparison of Predictive Values of Clinical Outcomes by Atlanta 1992, Atlanta 2012, and DBC Using ROC and Somer D

## DISCUSSION

According to our results, all the 3 classification systems accurately classify the severity of AP. However, the Atlanta 2012 and the DBC performed better than the Atlanta 1992, and they were comparable in predicting long-term clinical prognosis, major complications, and clinical interventions.

### Comparison With Previous Study

To compare the association between different severity classifications and clinical outcomes, several studies were recently published.^17–20^ In the 3 most recent studies,^17,18,20^ they all demonstrated that both the DBC and the Atlanta 2012 accurately classify the severity of AP in subgroups of patients. However, they did not study which one was better. Moreover, 2 previous studies enrolled relatively limited number of patients (N = 137^20^ and 151^18^), which could bring in some uncertainty to the conclusion. For the study by Acevedo-Piedra et al,^18^ although it enrolled as many as 532 patients, very few patients (N = 3) in their study suffered from critical AP.^17^ Nawaz et al^19^ used a similar method in our study to compare the association between the 3 different severity classifications and disease severity. They demonstrated that the Atlanta 2012 and the DBC accurately reflected clinical outcomes and were superior to the Atlanta 1992 evidenced by better predicting value in mortality (AUC 0.89 for both vs 0.76 for the Atlanta 1992, *P* < 0.001). But in their study, the DBC performed better than the Atlanta 2012 and the Atlanta 1992 in predicting need for interventions (*P* < 0.001), whereas the Atlanta 2012 performed better than the DBC and the Atlanta 1992 in predicting LOS (*P* < 0.05), which were different from our study. Importantly, relatively small number of patients in the moderate and critical groups in their study according to the DBC might lead to underpowered results during the pairwise comparisons between different DBC categories. For example, in their study, mortality was similar between severe and critical AP, which was totally different from the results of our study and a large recent meta-analysis.^21^

Our study enrolled more observational metrics and our study enrolled 395 patients with more reasonable and balanced distribution that could enhance the statistical power of our analysis. More patients in our study suffered from severe AP (42 [10.6%]) and critical AP (49 [12.4%]) according to the DBC. In our study, all the 3 classification systems were associated with statistically worse clinical outcomes. The Atlanta 2012 and the DBC performed better than the Atlanta 1992, and they were comparable in predicting long-term clinical prognosis, major complications, and clinical interventions.

In head-to-head comparison, the DBC appeared to perform better than the Atlanta 1992 in predicting the need for interventions. This may be driven by that IPN, a causal association with severity in the DBC, was a pivotal complication usually requiring surgical drainage.^22^ Moreover, it may also serve to induce sepsis and other complications. For the Atlanta 2012, it presumes only one “causal association” with severity, called persistent OF. It is easy for us to understand that patients with persistent OF need longer CRRT and/or MV therapy. However, it also performed better than Atlanta 1992, and it was comparable with the DBC in predicting surgical drainage. This could by driven by that patients with persistent OF were more likely to suffer from IPN.^2^ In our study 72% patients with persistent OF (severe AP according to the Atlanta 2012) suffered from IPN whereas only 11% patients developed IPN in the moderately severe AP group.

### Limitation of the Atlanta 2012 and the DBC Systems

**FIGURE 1:**
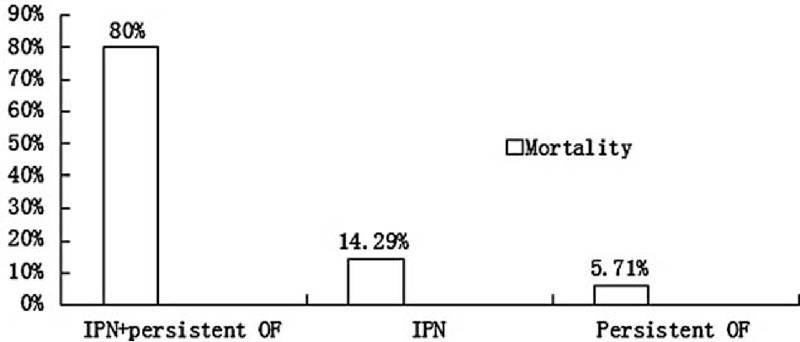
Distribution of hospital mortality when patients developed differential complications. IPN = infected pancreatic necrosis; persistent OF = organ failure ≥48 h.

Our study demonstrated that the Atlanta 2012 and the DBC were better than Atlanta 1992. However, both classification systems were with their limitations. For the Atlanta 2012, logistic regression test showed that IPN was significantly associated with hospital mortality (*P* < 0.001), therefore IPN should be a causal association with severity. Moreover, among the patients died in hospital, 80% died from both IPN and persistent OF (Figure 1); 14.29% died from IPN and uncontrolled infection; whereas the other 5.71% died from persistent MODS in the early stage of AP without detectable IPN. However, in this classification, patients with IPN and patients with acute fluid collection or others were assigned to the same group, which might be controversial.

For the DBC, 2 patients died from persistent OF in the early stage of AP without detectable IPN. For the 2 patients who died in the early stage of AP, how should we classify them as we got no opportunities to detect whether they would suffer from IPN or not. Moreover, the DBC defined only 2 “determinants,” PN and OF. They did not mention other potential “determinants” of severity, such as obesity,^13^ diabetes mellitus,^23^ and hypertriglyceridemia.^24^ Moreover, comorbidity is a well-known risk factor for mortality in AP and 4 patients died due to exacerbation of previous diseases (mainly heart diseases) in a previous study.^25^ Thus, the importance of other factors associated with severity needed to be assessed.

### Limitation of Our Study

One of the limitations of our study was that our SICU was a tertiary center, so a great part of patients in our study suffered from severe form of AP. We could not make sure whether this phenomenon might bring a bias to the study. Moreover, due to the functions of our SICU, our clinicians were likely to admit patients who were likely to suffer from severe form of AP according to their judgments, which could also potentially result in selection bias. However, this phenomenon did help us enroll more patients who suffered from severe form of AP when compared with other studies.

## CONCLUSION

All the 3 classification systems accurately classify the severity of AP. However, the Atlanta 2012 and DBC performed better than the Atlanta 1992, and they were comparable in predicting long-term clinical prognosis, major complications, and clinical interventions.
